# *Lactiplantibacillus plantarum* YS-718 Probiotics Screened from Traditional Chinese Fermented Vegetables for Aflatoxin B_1_ Removal

**DOI:** 10.3390/toxins18070275

**Published:** 2026-06-23

**Authors:** Fang Yuan, Guofeng Chen, Xianglong Yang, Ling Cheng, Qi Zhang, Peiwu Li, Baohai Liu, Jin Mao

**Affiliations:** 1 Key Laboratory of Quality and Safety of Cereals and Their Products, State Administration for Market Regulation, Quality and Safety Institute of Agricultural Products, Heilongjiang Academy of Agricultural Sciences, Harbin 150086, China; hljjiance@163.com; 2 National Reference Laboratory for Agricultural Testing, Key Laboratory of Detection for Mycotoxins, Laboratory of Quality & Safety Risk Assessment for Oilseed Products (Wuhan), Ministry of Agriculture and Rural Affairs, Oil Crops Research Institute, Chinese Academy of Agricultural Sciences, Wuhan 430062, China; yuanf202605@163.com (F.Y.); yangxianglong@caas.cn (X.Y.); chengling@cass.cn (L.C.); zhangqi01@caas.cn (Q.Z.); peiwuli@oilcrops.cn (P.L.)

**Keywords:** aflatoxin B_1_, fermentation probiotics, *Lactiplantibacillus plantarum*, removal

## Abstract

Aflatoxin contamination is the main risk factor in grain and oil crops, which brings serious threats to food and feed safety. Exploring a green and safe way to reduce aflatoxin is meaningful. In this study, six strains with aflatoxin removal ability are screened from traditional Chinese fermented vegetables. It was found that *Lactiplantibacillus plantarum* YS-718, as fermentation probiotics, showed the best performance on the aflatoxin B_1_ mitigation with the removal rate of 78.15% in liquid fermentation. To investigate the mechanism of removal, the aflatoxin B_1_ reduction tests by different components of *Lactiplantibacillus plantarum* YS-718 demonstrated that the bacterial suspension of *Lactiplantibacillus plantarum* YS-718 fermentation exhibited stronger adsorption ability compared to the removal ability of the supernatant of YS-718 fermentation. In addition, the *Lactiplantibacillus plantarum* YS-718 and aflatoxin B_1_ complex retained 43.74% of adsorption ability after four times repeated elution with PBS and 37.22% of adsorption after digestion with simulated gastric fluid for four hours. Moreover, *Lactiplantibacillus plantarum* YS-718 could be used to reduce aflatoxin B_1_ in peanut meal. By evaluating the contents of protein, amino acids, total sugars, and fatty acids after the fermentation treatment, it was found that *Lactiplantibacillus plantarum* YS-718 fermentation could increase the contents of protein, fatty acids, and amino acids in peanut meal. This study might provide useful information for constructing a green, safe, and efficient method for removing aflatoxin from peanut meal.

## 1. Introduction

Aflatoxins (AFs) are secondary metabolites produced primarily by *Aspergillus flavus* and *Aspergillus parasiticus* [1]. Over 20 naturally occurring aflatoxins have been identified, including aflatoxin B_1_ (AFB_1_), aflatoxin B_2_, aflatoxin G_1_, aflatoxin G_2_, aflatoxin M_1_, and aflatoxin M_2_, of which AFB_1_ is the most common and hazardous [2,3]. Aflatoxins possess carcinogenic, immunosuppressive, genotoxic, and mutagenic properties [4,5]. Exposure to or consumption of aflatoxin-contaminated food or feed can bring health threats to humans and animals, which can even lead to death [6]. Aflatoxins are commonly found in peanuts, maize, soybeans, rice, and other grain and oil crops [7]. Peanut meal is an important by-product of the peanut oil extraction process, with nearly 8 million tons of production annually worldwide [8]. Peanut meal is rich in protein, which could be used as the raw material in food processing and fermented feed. However, peanut meal protein contains allergenic components (*Ara h1* to *Ara h9*) that can be utilized as substrates for *Aspergillus flavus* and *Aspergillus parasiticus* [9]. In this case, aflatoxins could be produced by *Aspergillus flavus* and *Aspergillus parasiticus* contaminated peanut meal during the storage and transport processes, bringing serious risks to food and feed safety [10]. Therefore, exploring efficient, safe, and green ways to reduce aflatoxin contamination in feed or food is critical and significant.

A series of physical, chemical, and biological strategies has been used to reduce aflatoxins in feed. Physical methods mainly include absorption [11], heating treatment [12], irradiation, and ultraviolet (UV) light irradiation [13,14], etc. In general, chemical strategies are used to reduce aflatoxin by oxidizing treatments such as ozone and hydrogen peroxide [15,16], alkaline treatments such as sodium hydroxide and ammonia [17], acidic treatments such as formic and acetic acid [13], chlorinating treatments such as sodium hypochlorite and chlorine dioxide [18], and sodium bisulfite [19]. Biological methods mainly include microbial degradation and microbial adsorption [20], involving enzymatic degradation or microbial cell adsorption to reduce the toxicity and concentration of aflatoxins [21,22,23]. The biological methods are considered effective ways for aflatoxin degradation due to their safety, efficiency, and environmental friendliness. *Bacillus*, *Lactobacillus,* and *Aspergillus* spp., such as *Bacillus licheniformis* CFR1 [24], *Bacillus subtilis* BCC 42005 [25], *Lactobacillus plantarum* C88 [26], *Stenotrophomonas* sp. CW117 [27] and *Aspergillus niger* RAF106 [28] have been isolated to detoxify aflatoxins. And it was found that adsorption of aflatoxins by *Lactiplantibacillus* was mainly through polysaccharides (e.g., peptidoglycan and teichoic acid) on the cell wall [29]. In recent years, the probiotics-biosorbent has become a research hotspot for aflatoxin removal.

Following the Food and Agriculture Organization (FAO) and the World Health Organization (WHO), probiotics with many health and beneficial effects on the host have attracted much attention from the food and feed industry [30,31]. These nonpathogenic microorganisms can be incorporated into many types of products, for instance, foods, feeds, dietary supplements, and drugs. Probiotics have many advantages, such as inhibiting the growth of harmful pathogens, preserving intestinal homeostasis, and modulating the immune system [32,33]. Moreover, *Lactobacilluscasei* L30 [34], *Lactobacillus* spp. [35], *Lactobacillus kefir* [36], and *Lactobacillus rhamnosus* [37] have been found to show significant detoxification effects on aflatoxins. However, most of these strains might not be used in practical samples such as feed and food. Therefore, screening probiotics from food or feed that could be used to reduce aflatoxins in peanut meal is meaningful and challenging.

Herein, the strains with the ability to remove AFB_1_ were screened from traditional Chinese fermented foods from different areas by liquid fermentation using coumarin as the only carbon source, and the performance of reducing AFB_1_ from peanut meal was further evaluated. And then, the fermentation probiotics with the better removal efficiency were identified by 16S-rDNA sequences, and the removal mechanism of fermentation probiotics was proposed. Finally, the changes in the main quality in peanut meal were analyzed before and after fermentation.

## 2. Results

### 2.1. Screening and Isolation of Strains for AFB_1_ Removal

**Figure 1:**
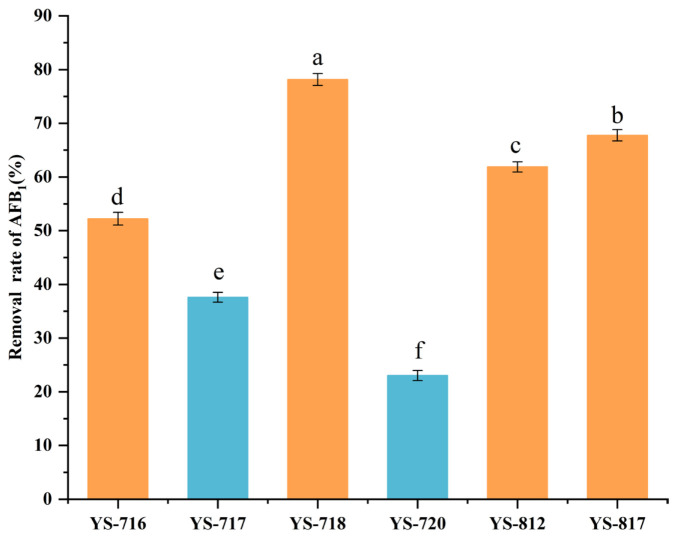
Removal rate of the primary screening strains after 96 h with AFB_1_ concentration of 30 ng/mL. Different letters indicate significant differences (*p* < 0.05).

Due to the aflatoxins’ structure, which contained the coumarin and dihydrofuran, it was beneficial to screen strains with aflatoxin reduction ability by using the coumarin as the carbon source [38]. In this study, six strains were screened by applying coumarin as the sole carbon source. To further verify the ability of the six initially screened strains to reduce AFB_1_, these six strains were rescreened using liquid fermentation. The amount of AFB_1_ residue after fermentation was detected by high-performance liquid chromatography (HPLC), and the AFB_1_ removal rate was calculated using Equation (3). As shown in Figure 1, all six strains had AFB_1_ removal ability, but at a primary concentration of 30 ng/mL, there was a difference in the AFB_1_ removal ability by different strains. The YS-718 strain had the highest rate of AFB_1_ removal, up to 78.15 ± 1.10%, which was higher than that of the other strains. YS-817, YS-812, and YS-716 were with removal rates of 67.76 ± 1.05%, 61.88 ± 0.93%, and 52.23 ± 1.19%, respectively, while YS-717 and YS-720 had a lower removal rate of 37.6 ± 0.92% and 23.03 ± 0.93%, respectively. Therefore, YS-718, YS-817, YS-812, and YS-716 were selected as candidate strains for AFB_1_ removal from peanut meal.

### 2.2. AFB_1_ Removal from Peanut Meal by Different Strains

**Figure 2:**
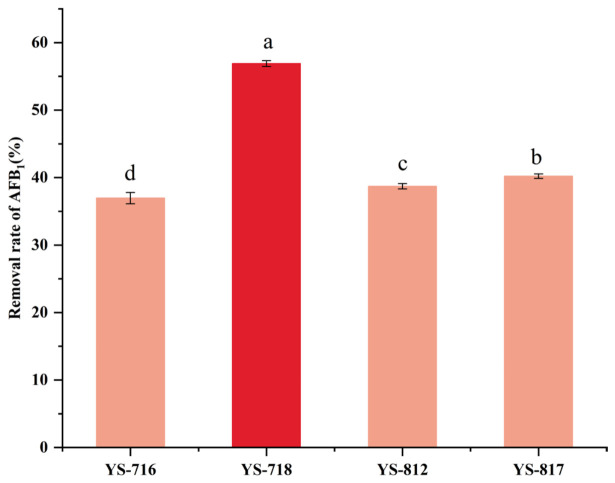
AFB_1_ removal by four strains in peanut meal fermentation. Different letters indicate significant differences (*p* < 0.05).

In order to select the strains more suitable for AFB_1_ removal in peanut meal, the above four strains obtained by liquid re-screening, as shown in Figure 2, were used to perform solid-state fermentation of peanut meal. After peanut meal fermentation by the YS-718 strain, the content of AFB_1_ in peanut meal was the lowest (12.85 ± 0.15 ng/g), and the removal rate was 56.89 ± 0.43% in peanut meal. Based on the above results and discussions, it could be found that the YS-718 strain had the highest removal rate of AFB_1_ in peanut meal, which was selected for further investigation.

### 2.3. Identification of YS-718 Strain

Initial examinations for purity and colony morphology served as the basis for the identification of the YS-718. The YS-718 strain was characterized by a cream-white, shiny appearance, exhibiting opaque, rounded colonies on MRS agar medium. These colonies that exhibited regular edges and a prominent center were easily picked up.

The total DNA of the YS-718 strain was extracted and amplified by PCR using bacterial 16S rDNA universal primers. The amplified products were sequenced, and the NCBI database homology analysis showed that the homology between the YS-718 strain and *Lactiplantibacillus plantarum* (KR153313.1) was 100%. It was identified as *Lactiplantibacillus plantarum* and named *Lactiplantibacillus plantarum* YS-718, and the information of YS-718 could be seen in Table S1 of the Supplementary Materials.

### 2.4. AFB_1_ Removal by Different Components of YS-718 Liquid Fermentation

To investigate the key components for AFB_1_ removal by YS-718, different components were isolated from the fermentation, and AFB_1_ content was detected after mixing these components with the AFB_1_ solution. Table 1 showed that LF–B had the higher removal rate of aflatoxin B_1_ with 78.15 ± 1.10%, while the initial concentration of AFB_1_ was 30 ng/mL. And LF-BS had a removal rate of 66.31 ± 0.79%, which was the closest to LF–B, while LF–S had a lower removal rate of AFB_1_ at 22.82 ± 1.34%. This indicated that the main component in the fermentation broth to reduce AFB_1_ was the bacterial suspension, while the supernatant showed less ability to remove AFB_1_.

### 2.5. Stability Study of YS-718 Strain Removal

#### 2.5.1. Effect of PBS Elution on the Adsorption Stability of YS-718 Strain

**Figure 3:**
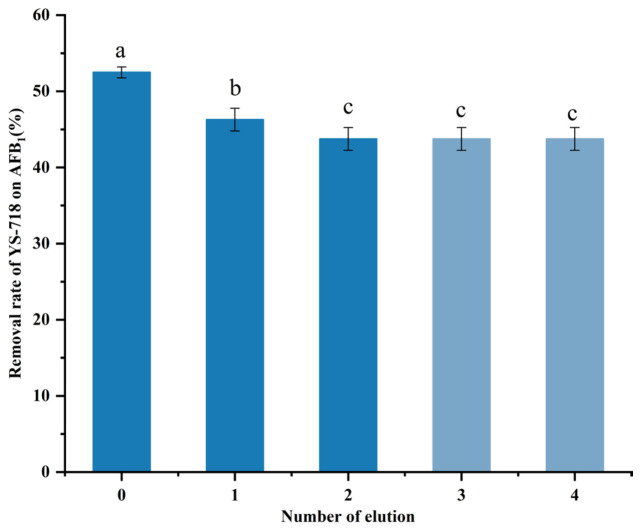
Effect of PBS elution on YS-718 and AFB_1_ complex. Different letters indicate significant differences (*p* < 0.05).

The adsorption stability of AFB_1_ by YS-718 was evaluated by repeatedly washing the YS-718 and AFB_1_ complexes with PBS and detecting the contents of AFB_1_ released by YS-718 after elution. Figure 3 showed that the highest release of AFB_1_ was observed during the first elution, decreasing from an initial adsorption of 52.48 ± 0.83% to 46.28 ± 1.75%. The release of AFB_1_ by YS-718 and AFB_1_ complexes tended to stabilize after twice PBS elutions, and after the third elution, and AFB_1_ was hardly detected in the eluate. This indicated that the adsorption of AFB_1_ by YS-718 was a reversible process, which is consistent with the results of Mosallaie [39].

#### 2.5.2. Effects of Simulated In Vitro Gastric Digestion on the Adsorption Stability of YS-718

**Figure 4:**
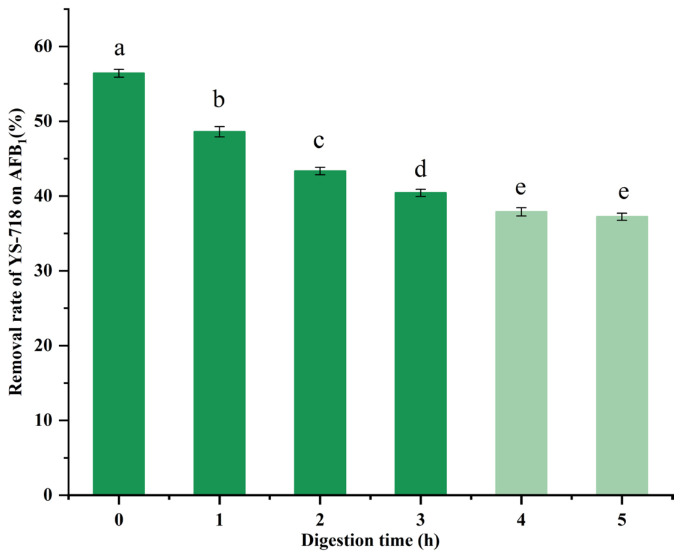
Effect of gastric juice digestion on YS-718 and AFB_1_ complex. Different letters indicate significant differences (*p* < 0.05).

In vitro simulated gastric fluid elimination was used to investigate the effect of gastric juice on the stability of the YS-718 and AFB_1_ complex and to assess its safety properties after consumption. Figure 4 showed that the YS-718 and AFB_1_ complex had the highest AFB_1_ release during the first hour of simulated in vitro gastric juice digestion. After 4~5 h of digestion, the amount of AFB_1_ released by YS-718 and the AFB_1_ complex tended to be stable. There was no significant difference in the retention adsorption rate, which was 37.22 ± 0.66% after 5 h of gastric juice digestion, accounting for 65.98 ± 0.71% of the initial adsorption amount. This illustrated that the fermented probiotic YS-718 had good stability and retained high adsorption rates after PBS and simulated gastric fluid elution.

### 2.6. Quality Analysis of Peanut Meal Before and After YS-718 Fermentation

Peanut meal can be used as a feed source or processed into other functional protein foods. Therefore, the nutritional value of peanut meal should be maintained as much as possible during AFB_1_ removal. The protein, amino acid, total sugar, and fatty acid contents of peanut meal were analyzed before and after fermentation to investigate the effect of YS-718 treatment on the peanut meal qualities. And it was found that the form and condition were not obviously changed after fermentation, as shown in Figure S2 of Supplementary Materials.

As shown in Table 2, the protein content of peanut meal after fermentation treatment was 44.49 g/100 g, which was 0.67 g/100 g higher than that before fermentation. The total sugar content was 170.95 ± 1.8 mg/g, which was 3.61% lower than that before fermentation. This might be attributed to the fact that YS-718 consumed part of the sugars by metabolism in the peanut meal. Moreover, the total fatty acid and unsaturated fatty acids increased simultaneously after the YS-718 fermentation, which might be due to the decreased absolute quantification of total sugar and an increase in the relative quantification of fatty acids. As we know, unsaturated fatty acids are indispensable fatty acids for animals, some of which cannot be synthesized by themselves and must be supplemented from the diet. This shows that the nutritional value has improved after the YS-718 fermentation. The effect of fermentation treatment on amino acids was also investigated and shown in Table 3. It could be found that most of the amino acids increased after fermentation, and total amino acids added from 40.46 g/100 g to 41.11 g/100 g, indicating that the nutritional value had increased.

## 3. Discussion

### 3.1. Possible Mechanism of AFB_1_ Removal by YS-718 Liquid Fermentation

Markov’s study showed that the removal of aflatoxins by bacterial suspension was mainly ascribed to adsorption by the bacterium [40]. Pourmohammadi et al. further demonstrated that bacterial adsorption was related to the cell wall [41], and Abrunhosa et al. showed that peptidoglycan or compounds closely bound to peptidoglycan were mainly involved in adsorption on the cell wall [42]. Moreover, it was found that the increase in AFB_1_ removal rate after heat treatment of the supernatant was consistent with the results of Sangare and Shu [43,44], which may be due to the thermal stability of the components in the supernatant involved in aflatoxin removal.

Compared LF–heated BS with LF–BS, it was found that the heat-treated suspension had a higher AFB_1_ removal rate of 71.21%. These results indicated that heat treatment could enhance the removal efficiency of AFB_1_ from bacterial suspension. Haskard et al. also found that the AFB_1_ adsorption ability of the strain was enhanced after heat treatment [21]. This heat treatment denatured the cell surface protein of the strain, so that more binding areas were exposed to AFB_1_ [35]. And Bueno et al. studied the ability of 12 *Lactobacillus* species to adsorb AFB_1_. The results showed that the aflatoxin reduction rate of different lactic acid bacteria strains was 25~61%. After washing the lactic acid bacteria adsorbing AFB_1_ with phosphate buffer and acetonitrile 5 times, 10%~50% of AFB_1_ was still adsorbed on the lactic acid bacteria. This might be due to the fact that the stability of the strain and AFB_1_ complex depended on the binding capacity of the strain itself and the strain-specific [45]. Therefore, the present experiments showed that YS-718 mitigated the AFB_1_ mainly through the adsorption by the bacterial suspension. However, the adsorption can not change the structure of AFB_1_, indicating that the toxicity site of AFB_1_ might not be changed. And the evaluation by repeatedly washing the YS-718 and AFB_1_ complexes with PBS showed that AFB_1_ can be released from the complex. This might affect its practical application in the digestive system, which needs a more in-depth in vivo study.

### 3.2. Quality Improvement of Peanut Meal After YS-718 Fermentation

Yang also found that the protein content of peanut meal was significantly increased by the fermentation of *Bacillus licheniformis* [46], which was mainly due to the respiration of the bacterium in fermentation, consuming part of the organic matter (releasing CO_2_ and H_2_O). A fraction of the increased proteins may also be those contained in microorganisms. The content of unsaturated fatty acids in peanut meal was increased 33.08% after fermentation, probably because the lipase produced by YS-718 breaks down the fat in peanut meal into fatty acids. The changes in amino acid content are shown in Table 3. The total amino acid content and essential amino acid content of peanut meal after fermentation were 41.11 ± 0.09 mg/g and 12.18 ± 0.01 mg/g, respectively, increased by 1.61% and 2.18% compared with those before fermentation, which were consistent with Li [47]. Among them, threonine, lysine, and histidine increased by 2.70%, 7.75%, and 4.44%, respectively. These results indicated that fermentation of AFB_1_ in detoxified peanut meal by YS-718 might improve its feeding value.

### 3.3. Future Study and Directions

This study screened *Lactiplantibacillus plantarum* YS-718 as a kind of probiotic to remove AFB_1_ in peanut meal and improved the nutritional value of peanut meal after fermentation, which provided a potential way to mitigate the risk of peanut meal contaminated by aflatoxins. However, deeper research might be carried out to identify the clearer mechanism of YS-718, such as the specific cellular component which played the main adsorption role, and the sites where the specific cellular component interacts with AFB_1_. And it was found that several kinds of proteins were separated by electrophoresis in the supernatant after YS-718 fermentation. Although the reduction efficiency of AFB_1_ by separated proteins was low (<5%), previous studies showed that detoxification of aflatoxins by strain supernatant might be achieved by enzymatic degradation [48,49,50,51]. It was also meaningful that the future study might focus on the specific components, such as the enzyme, to study the degradation mechanism.

Based on the findings and challenges of this study, future research should focus on the following directions: characterizing the interaction between YS-718 cellular components and aflatoxins to clarify the role of cell membranes, cell walls, etc.; utilizing infrared spectroscopy, elemental analysis, and theoretical calculations to elucidate the chemical binding site of the specific component in cell membranes or cell walls interacting with AFB_1_; exploring the active substances in the YS-718 supernatant through the advanced separation methods and high-resolution mass spectrometry; and identifying the structure of active substances. In this study, the peanut meal was sterilized at 121 °C for 20 min. However, for the practical application, the YS-718 bacterial suspension in peanut meal will raise the moisture levels, which might promote the growth of various strains or mycotoxigenic fungi. In order to control the risk of these concerns, it was necessary to detect the change in microbial populations in the peanut meal during the fermentation. And the efficiency of YS-718 should also be improved by the strain domestication to shorten the 96 h fermentation time. YS-718 was deposited in the Chinese Typical Cultures Depository Center (CCTCC) with the accession number CCTCC M 20232226, and it was found that the lyophilization capacity, storage stability, and survival after drying of YS-718 were well maintained through cultural activation again. Therefore, these deeper studies were meaningful and challenging, which will promote the practical application of *Lactiplantibacillus plantarum* YS-718 probiotics.

## 4. Conclusions

In conclusion, *Lactiplantibacillus plantarum* YS-718 screened from Chinese traditional fermented vegetables showed the well removal efficiency of AFB_1_ in peanut meal. And it could also improve the protein, fatty acid, and amino acid contents of peanut meal after treatment. And it was found that the strain mainly reduces the AFB_1_ through adsorption, and the adsorption ability was also stable after repeated elution with PBS or in vitro simulation of gastric fluid digestion. The *Lactiplantibacillus plantarum* YS-718 not only can mitigate AFB_1_ in peanut meal but also improves the nutritional value of peanut meal after fermentation, which makes it can be regarded as a potential candidate for aflatoxin removal in practical applications. Most importantly, research is underway to improve the efficiency of aflatoxin removal through *Lactiplantibacillus plantarum* YS-718 gene editing and domestication.

## 5. Materials and Methods

### 5.1. Chemicals and Reagents

A total of 1 mg AFB_1_ standard (99.9% purity, Sigma Chemical Co., St. Louis, MO, USA) was dissolved with acetonitrile to form a stock solution and stored at −20 °C.

Peanut meal samples were purchased from the market (Wuhan, China). MRS agar medium (MRS ager:1% peptone, beef extract power 0.8%, 0.4% yeast extract power, 2% glucose, 0.2% dipotassium hydrogen phosphate, 0.2% diammonium hydrogen citrate, 0.5% sodium acetate, 0.02% magnesium sulfate, 0.004% manganese sulfate, 0.1% tween 80 and 1.4% agar) and MRS Broth medium (MRS broth:1% peptone, beef extract power 0.8%, 0.4% yeast extract power, 2% glucose, 0.2% dipotassium hydrogen phosphate, 0.2% diammonium hydrogen citrate, 0.5% sodium acetate, 0.02% magnesium sulfate, 0.004% manganese sulfate) were purchased from Qingdao Haibo Biotechnology Co., Ltd. (Qingdao, China), and other chemicals such as KNO_3_, KH_2_PO4, MgSO_4_‧7H_2_O, (NH4)_2_SO_4_, and FeCl_3_‧6H_2_O were purchased from Sinopharm Group (Shanghai, China); CaCl_2_‧2H_2_O, pepsin and coumarin were purchased from Aladdin Biochemical Technology Co., Ltd. (Shanghai, China); agar was purchased from Biofroxx Co., Ltd. (Munich, Germany).

### 5.2. Screening and Isolation of Strains for AFB_1_ Removal

Traditional Chinese fermented vegetables are a natural strain source. Two kinds of traditional fermented vegetables were collected from markets in Liuzhou City, Guangxi Province, and Dingxi City, Gansu Province. A 5.00 g fermented vegetables sample was put into a sterile tube, and 45 mL of MRS Broth was added. After being fully shaken on a spiral device at 37 °C for 12 h, the liquid supernatant was diluted to 10^−2^, 10^−3^, 10^−4^, 10^−5^, or 10^−6^, and then 100 µL diluent was spread onto the MRS agar medium. After that, all the samples were incubated in an incubator at 37 °C for 2–3 days to observe the growth of the colonies on the Petri dishes. A single colony with good growth conditions was selected and transferred to coumarin medium (CM: 0.05% KNO_3_, 0.025% KH_2_PO_4_, 0.025% MgSO_4_‧7H_2_O, 0.05% (NH_4_)_2_SO_4_, 0.0003% FeCl_3_‧6H_2_O, and 0.005% CaCl_2_‧2H_2_O, 2% agar, and 0.1% coumarin). And then a single colony with good growth conditions was selected and transferred to MRS agar medium, which was purified several times to obtain pure cultures. It was found that strains with AFB_1_ removal potential could be screened using coumarin as the sole carbon source [38]. Six morphologically different strains shown in Figure S1 of Supplementary Materials, noted YS-716, YS-717, YS-718, YS-720, YS-812, and YS-817, were primarily screened and isolated from traditional fermented vegetables with different origins by using coumarin as the sole carbon source of the medium. It was found that these strains were pure and the surface was smooth.

To detect the removal efficiency of AFB_1_ by the collected strains, single colony was picked out from the above MRS agar medium and inoculated into MRS broth medium, which was then incubated on a shaker at 37 °C and 200 r/min for 12 h to obtain the fermentation broth. Then, 30 μL of 1000 ng/mL AFB_1_ standard solution was added to 970 μL of fermentation broth (5.0 ×10^8^ CFU/mL) to achieve a final AFB_1_ concentration of 30 ng/mL. The mixture was then incubated in a constant temperature shaker at 37 °C and 200 r/min for 96 h. Finally, the AFB_1_ content was extracted and detected by the method in Section 5.7.

### 5.3. AFB_1_ Removal in Peanut Meal by Different Strains and Strains Identification

To evaluate the AFB_1_ removal efficiency in peanut meal during fermentation, the obtained strains from traditional Chinese fermented vegetables were tested. Firstly, 3 mL of sterile water and 150 μL of AFB_1_ standard solution (1000 ng/mL) were added to 5.00 g of sterile peanut meal (sterilized at 121 °C for 20 min), and then 1 mL of fermentation broth of the four strains at a cell concentration of 5.0 × 10^8^ CFU/mL was added, respectively, and incubated for 96 h at 37 °C. All experiments were repeated three times under the same conditions. AFB_1_ extraction and detection are described in Section 5.7. The isolated strains were identified by morphology and were confirmed by 16S rDNA basal phylogenetic analysis. The purified strains were sent to Tsingke Biotechnology Co., Ltd (Beijing, China). for 16S rDNA sequencing. The 16S rDNA gene sequences of the strains were compared at the National Center for Biotechnology Information (NCBI) using Blast, and those with homologies >98% were considered as the same species.

### 5.4. AFB_1_ Removal by Different Components of YS-718 Liquid Fermentation

YS-718 was cultured in MRS broth at 37 °C and 200 r/min for 12 h until the cell concentration reached approximately 5 × 10^8^ CFU/mL to obtain the YS-718 fermentation broth (LF-B). The fermentation broth was centrifuged at 5000 r/min and 4 °C for 10 min, then the upper liquid was filtered through a 0.22 μm sterile filter membrane to obtain the supernatant of YS-718 fermentation broth (LF-S). The centrifuged precipitate was washed three times with phosphate-buffered solution (PBS, 0.01 moL/L, pH 7.4) and re-suspended in PBS to obtain the original cell concentration of bacterial suspension (LF-BS). The fermentation broth, extracellular supernatant, and bacterial suspension were bathed in water at 98 ± 1 °C for 30 min to obtain heat-treated fermentation broth (LF-heated B), heat-treated supernatant (LF-heated S), and heat-treated bacterial suspension (LF-heated BS). AFB_1_ was added to the LF-B, LF-ES, LF-BS, LF-heated B, LF-heated ES, and LF-heated BS at a final concentration of 30 ng/mL, respectively. The various treatments were then incubated on an unilluminated rotary shaker (200 r/min, 37 °C) for 96 h. All experiments were repeated three times under the same conditions. AFB_1_ extraction and detection are described in Section 5.7.2 and Section 5.7.3.

### 5.5. Stability Studies of the YS-718-AFB_1_ Complex

According to the research of Hernandez-Mendoza [34], most of the AFB_1_ removal mechanisms of *Lactobacillus* and *Saccharomyces* are to utilize different components of the cell wall to adsorb AFB_1_ and form a bacterium and AFB_1_ complex, which can affect the removal. The adsorption of AFB_1_ by strains is connected by non-covalent bonds, and the stability of adsorption is determined by strain specificity. The adsorption stability of YS-718 was investigated by elution with PBS and in vitro digestion with simulated gastric fluid.

YS-718 was followed by centrifugation to obtain the precipitate, which was selected for the removal test. Subsequently, the precipitate was resuspended in an equal volume of PBS solution (pH 7.4) and incubated at 37 °C for 30 min. After centrifugation at 5000 rpm for 15 min, the supernatant was collected. The above process was repeated four times, and the supernatant was collected cumulatively. All experiments were repeated three times under the same conditions. AFB_1_ extraction and detection are described in Section 5.7. The stability of the YS-718 removal ability was calculated by Equation (1).

(1) $$M\%=\left(1-\frac{P_{1}}{P_{2}}\right)\times 100$$

where M: strain retention adsorption rate, %; P_1_: AFB_1_ content in the cumulative supernatant, ng/mL; P_2_: complex AFB_1_ content of the initial strain, ng/mL.

YS-718 underwent a removal test, followed by centrifugation to obtain precipitate. Subsequently, the precipitate was resuspended in an equal volume of gastric fluid simulants (Simulant: 16.4 mL of dilute hydrochloric acid with about 800 mL of water and 10 g of pepsin, mix well, and then add water to a volume of 1000 mL) [52]. The mixture was then incubated on a shaker at 37 °C and 200 rpm for 5 h, and the supernatant was collected cumulatively at 1 h intervals. All experiments were repeated three times under the same conditions. AFB_1_ extraction and detection are described in Section 5.7.2 and Section 5.7.3. The stability of the YS-718 adsorption ability was calculated by Equation (1).

### 5.6. Evaluation of Peanut Meal Quality Before and After Fermentation

The effect of fermentation treatment on the quality of peanut meal was investigated by determining the protein content, amino acid content, total sugar content, and fatty acid content of peanut meal before and after fermentation with YS-718. These quality indices of peanut meal were detected before the fermentation and after the addition of YS-718 for 96 h, respectively.

#### 5.6.1. Protein Content

According to the China National Standard, GB 5009.5-2016 [53], the Kjeldahl method was modified to determine the protein content in peanut meal. Firstly, a 0.20 g peanut meal sample was weighed (marked as m) and placed in a digestive tube. Then, 8 mL of sulfuric acid and a mixed tablet containing copper sulfate and potassium sulfate were added to the digestive tube, which was subsequently placed in a digestion furnace and digested at 420 °C for 2.5 h until the liquid was green and transparent. The blank experiment and sample experiment were carried out simultaneously. After cooling, the liquid was placed in a K1160 automatic Kjeldahl nitrogen analyzer (Haineng Future Technology Group Co., Ltd., Shandong, China). Sodium hydroxide solution, standard hydrochloric acid solution, and the boric acid solution containing mixed indicator were added before determining the protein content.

(2) $$X\%=\frac{\left(V_{1}-V_{2}\right)\times c\times 0.0140\times 5.46\times 100}{m\times V_{3}/100}$$

where V_1_: Volume of standard titrant of hydrochloric acid consumed in the treatment group, mL; V_2_: Volume of standard titration solution of hydrochloric acid consumed in the blank group, mL; V_3_: Volume of the digestion solution, mL; c: Concentration of standard titration solution of hydrochloric acid, moL/L; 0.0140: The hydrochloric acid standard titration solution is equivalent to the mass of nitrogen, g; m: Mass of the sample, g; 5.46: Coefficient for the conversion of nitrogen to protein in peanut meal; 100: Conversion coefficient.

#### 5.6.2. Amino Acid Content

According to the Chinese national standard GB 5009.124-2016 [54], the ninhydrin post-column derivatization ion-exchange chromatography (IEC) method was improved for the determination of amino acid content in peanut meal. First, 0.50 g of the peanut meal sample was weighed (marked as m) and placed in a hydrolysis tube. Next, 15 mL of 6 moL/L hydrochloric acid was added to the hydrolysis tube. The tube was evacuated to near 0 Pa with a vacuum pump, and filled with nitrogen, which was repeated three times, and then sealed under the nitrogen-filled condition. The sealed hydrolysis tubes were placed in an electric blast thermostat at 110 ± 1 °C for 22 h. After cooling, the hydrolysate was filtered into a 50 mL volumetric flask, adjusted to the scale with water, and shaken well, then filtered through a 0.22 μm membrane. After aspirating 200 μL of filtrate and freeze-drying for 5 h, 1 mL of 0.02 moL/L hydrochloric acid was added to re-dissolve the solution, which was finally analyzed by LA8080 Amino acid autoanalyzer. (Shanghai Hitachi High-Technologies Co., Ltd., Shanghai, China) for analysis.

#### 5.6.3. Total Sugar Content

The total sugar content in peanut meal was determined by a Total Sugar Content Assay Kit AKSCU003C (Beijing Boxbio Science & Technology Co., Ltd., Beijing, China).

#### 5.6.4. Fatty Acid Content

Fatty acid content was determined by the previous analytical method [55]. The specific sample preparation processes are as follows: firstly, weigh 0.30 g–0.50 g of the peanut meal sample in a 10 mL centrifuge tube, and then 2 mL diethyl ether/petroleum ether (50:50 *v*/*v*) was added. The dilution was then mixed with 1 mL of 0.4 mol/L KOH-CH_3_OH, and after vortexing, stood for reaction for 2.5 h at room temperature. It was then vortexed and given 2 mL of ultra-pure water. For stratification, the solution was set aside overnight. At last, 200 μL of supernatant was taken, and 800 μL of petroleum ether was added to obtain the sample solution to be tested. Analysis was performed with Agilent 7890 A gas chromatography (Agilent, Palo Alto, CA, USA) equipped with a flame ionization detector (FID) (Thermo Fisher, MA, USA) and a capillary column Agilent J & W 123–2332 (0.25 μm, 60 m 0.25 mm, Agilent, Waltham, MA, USA). Operating conditions were as follows: the oven temperature was initially set at 180 °C for 2 min, then raised to 220 °C at 3 °C/min and held isothermal for 12 min. The injection volume was 1 μL using a 250 °C injector temperature at a 1:80 split ratio. The carrier gas was high-purity nitrogen at 25 mL/min. The retention times of the spectra of the samples to be tested were compared with those of the standard samples, and the external standard method was used for quantification.

### 5.7. Extraction and Detection of AFB_1_

#### 5.7.1. Extraction of AFB_1_ from Samples

AFB_1_ extraction and detection were executed and modified according to the previous method [39,56]. AFB_1_ was extracted as follows: 5.00 g samples were weighed into a centrifuge tube. After adding 25 mL acetonitrile/water (84:16, *v*/*v*) and mixing for 10 min, the samples were centrifuged at 5000 rpm for 15 min at room temperature. A total of 1 mL of supernatant was diluted with 5 mL PBS, and then 6 mL of the diluted sample was extracted with a homemade immunoaffinity column, eluted with 1 mL of methanol. Finally, the eluate was collected and filtered through a 0.22 µm organic membrane, and the filtrate was collected into a sample vial.

#### 5.7.2. Extraction of AFB_1_ from Liquids

An equal volume of dichloromethane was added to the culture broth that had completed fermentation, shaken and mixed thoroughly, then left to stratify. Collect the lower layer of dichloromethane and extract three times. The collected methylene chloride was blown with nitrogen in a water bath at 40 °C until no liquid remained. It was redissolved in 1 mL of methanol and filtered through a 0.22 µm organic membrane, and the filtrate was collected for testing.

#### 5.7.3. Detection of AFB_1_

The detection of AFB_1_ was as follows: high-performance liquid chromatography (HPLC) with post-column photochemical derivatization reactor under 254 nm ultraviolet irradiation (AURA PHRED, CA, USA) detect AFB_1_ in the supernatant. The excitation and detection wavelengths were set at 360 nm and 440 nm, respectively. The removal rate (A) was calculated by Equation (3).

(3) $$A\%=\left(1-\frac{c_{1}}{c_{2}}\right)\times 100$$

where c_1_: concentration of AFB_1_ in the sample; c_2_: concentration of AFB1 in the control. AFB_1_ was analyzed according to retention time in the HPLC system. The samples were separated by HPLC (Wooking K2025, Shandong Wooking Technology Co., Ltd., DeZhou, China) equipped with a ZORBAX Eclipse XDB-C18 column (4.6 × 150 mm, 5 µm, Agilent, Palo Alto, CA, USA) and a 470-fluorescence detector (RF-20A, Shimadzu, Kyoto, Japan) (λexc 360 nm; λem 440 nm) using a mobile phase solvent of 45% methanol and 55% water. The flow rate was 0.8 mL/min, and the injection volume was 10 µL.

Results were statistically compared and expressed as means with standard deviations (SD). Data were processed by Excel 2019 and SPSS 26.0, significant differences were analyzed by ANOVA and Duncan test (*p* < 0.05), and graphical analysis was performed by Origin 2021.

## Figures and Tables

**Table 1:** AFB_1_ removal ability by different components of YS-718.

Item	Concentration of AFB_1_ (ng/mL) in Solution After Removal	Removal Ratio (% of Control)
LF–B	6.51 ± 0.33	78.15 ± 1.10 b
LF–heated B	5.70 ± 0.20	80.86 ± 0.67 a
LF–S	23.00 ± 0.40	22.82 ± 1.34 f
LF–heated S	20.15 ± 0.16	32.37 ± 0.53 e
LF–BS	10.03 ± 0.23	66.31 ± 0.79 d
LF–heated BS	8.58 ± 0.28	71.21 ± 0.88 c

Note: The initial concentration of AFB_1_ was 30 ng/mL. LF–B (YS-718 fermentation broth), LF–heated B (YS-718 fermentation broth after heat treatment), LF–S (the supernatant of YS-718 fermentation broth by centrifugation), LF–heated S (the supernatant of YS-718 fermentation broth by centrifugation after heat treatment), LF–BS (the precipitate or suspension of YS-718 fermentation broth by centrifugation, which is resuspended in sterile PBS), and LF–heated BS (the precipitate or suspension of YS-718 fermentation broth by centrifugation after heat treatment). Different letters indicate significant differences (*p* < 0.05).

**Table 2:** The quality analysis of peanut meal samples before and after fermentation.

Peanut Meal	Protein (g/100 g)	Total Sugar (mg/g)	Total Fatty Acid (%)	Unsaturated Fatty Acid (%)
Before fermentation	44.49 ± 0.15 b	177.36 ± 0.20 a	1.41 ± 0.22 b	1.33 ± 0.20 b
After fermentation	45.16 ± 0.02 a	170.95 ± 0.21 b	1.87 ± 0.20 a	1.77 ± 0.14 a

Notes: Control represented the peanut meal without fermentation. Different letters indicate significant differences (*p* < 0.05).

**Table 3:** The content of amino acids in peanut meal before and after fermentation.

Amino Acid Type	Before Fermentation (g/100 g)	After Fermentation (g/100 g)
Aspartic acid (Asp)	5.09 ± 0.05	5.17 ± 0.02
Glutamic acid (Glu)	1.11 ± 0.02	1.14 ± 0.02
Serine (Ser)	2.07 ± 0.07	2.12 ± 0.01
Histidine (His)	8.60 ± 0.14	8.76 ± 0.01
Glycine (Gly)	2.41 ± 0.02	2.46 ± 0.01
Threonine * (Thr)	1.69 ± 0.02	1.74 ± 0.02
Arginine (Arg)	0.54 ± 0.03	0.55 ± 0.02
Alanine (Ala)	1.72 ± 0.01	1.74 ± 0.01
Tyrosine (Tyr)	0.29 ± 0.04	0.24 ± 0.01
Cysteine (Cys-s)	1.44 ± 0.01	1.45 ± 0.01
Valine * (Val)	2.85 ± 0.02	2.90 ± 0.01
Methionine * (Met)	1.41 ±0.02	1.37 ± 0.02
Phenylalanine * (Phe)	2.20 ± 0.02	2.23 ± 0.01
Isoleucine * (Ile)	1.42 ± 0.02	1.53 ± 0.01
Leucine * (Leu)	0.90 ±0.00	0.94 ± 0.01
Lysine * (Lys)	4.93 ± 0.06	4.89 ± 0.03
Proline (Pro)	1.81 ± 0.02	1.86 ± 0.02
Total essential amino acids	11.92 ± 0.06	12.18 ± 0.01
Total Amino Acids	40.46 ± 0.44	41.11 ± 0.09

Notes: * Represents essential amino acids.

## Data Availability

Data is contained within the article or Supplementary Materials.

## Supplementary Materials

The following supporting information can be downloaded at: https://www.mdpi.com/article/10.3390/toxins18070275/s1. Figure S1: The photographs of the six screened strains using coumarin as the sole carbon source. Figure S2. The photographs of peanut meal samples before and after treatment/fermentation. Table S1: The identification information of *Lactiplantibacillus plantarum* YS-718.

## References

[1] Bhardwaj K., Meneely J.P., Haughey S.A., Dean M., Wall P., Zhang G., Baker B., Elliott C.T. (2023). Risk assessments for the dietary intake aflatoxins in food: A systematic review (2016–2022). Food Control.

[2] Jaćević V., Dumanović J., Alomar S.Y., Resanović R., Milovanović Z., Nepovimova E., Wu Q., Franca T.C.C., Wu W., Kuča K. (2023). Research update on aflatoxins toxicity, metabolism, distribution, and detection: A concise overview. Toxicology.

[3] Wang S., Herrera-Balandrano D., Shi X., Chen X., Liu F., Laborda P. (2023). Occurrence of aflatoxins in water and decontamination strategies: A review. Water Res..

[4] Dai H.R., Liang S.H., Shan D.D., Zhang Q.P., Li J., Xu Q., Wang C.M. (2022). Efficient and simple simultaneous adsorption removal of multiple aflatoxins from various liquid foods. Food Chem..

[5] Khan R., Ghazali F.M., Mahyudin N.A., Samsudin N.I.P. (2021). Aflatoxin Biosynthesis, Genetic Regulation, Toxicity, and Control Strategies: A Review. J. Fungi.

[6] Okechukwu V.O., Adelusi O.A., Kappo A.P., Njobeh P.B., Mamo M.A. (2023). Aflatoxins: Occurrence, Biosynthesis, Mechanism of Action and Effects, Conventional/Emerging Detection Techniques. Food Chem..

[7] Wang X., Wang T., Nepovimova E., Long M., Wu W., Kuca K. (2022). Progress on the detoxification of aflatoxin B_1_ using natural anti-oxidants. Food Chem. Toxicol..

[8] Chen Y., Fang Q., Zhang L., Zhang K., Wei T., Ping Q. (2021). Directional production of even carbon volatile fatty acids from peanut meal: Effects of initial pH and hydraulic residence time. Environ. Eng. Res..

[9] Zhao T., Ying P., Zhang Y., Chen H., Yang X. (2023). Research Advances in the High-Value Utilization of Peanut Meal Resources and Its Hydrolysates: A Review. Molecules.

[10] Hariharan S., Patti A., Arora A. (2023). Functional Proteins from Biovalorization of Peanut Meal: Advances in Process Technology and Applications. Plant Foods Hum. Nutr..

[11] Ma F., Cai X., Mao J., Yu L., Li P. (2021). Adsorptive removal of aflatoxin B_1_ from vegetable oils *via* novel adsorbents derived from a metal-organic framework. J. Hazard. Mater..

[12] Mahoney N.E., Cheng L., Palumbo J.D. (2020). Effect of Blanching on Aflatoxin Contamination and Cross-Contamination of Almonds. J. Food Prot..

[13] Babaee R., Karami-Osboo R., Mirabolfathy M. (2022). Evaluation of the use of Ozone, UV-C and Citric acid in reducing aflatoxins in pistachio nut. J. Food Compos. Anal..

[14] Thimuthu Kasun B.V., Vanniarachchy M.P.G. (2023). Reduction of Aflatoxin contamination in coconut oil using concentrated solar radiation. Food Chem. Adv..

[15] Kamber U., Gülbaz G., Aksu P., Doğan A. (2017). Detoxification of Aflatoxin B1 in Red Pepper (*Capsicum annuum* L.) by Ozone Treatment and Its Effect on Microbiological and Sensory Quality. J. Food Process. Preserv..

[16] Nesci A., Passone M.A., Barra P., Girardi N., García D., Etcheverry M. (2016). Prevention of aflatoxin contamination in stored grains using chemical strategies. Curr. Opin. Food Sci..

[17] Thakaew R., Chaiklangmuang S. (2023). Aflatoxin B_1_ elimination in low-grade maize by co-influence of heat and chemical treatment. Qual. Assur. Saf. Crops Foods.

[18] Liu Z., Cao Z., Wang J., Sun B. (2022). Chlorine dioxide fumigation: An effective technology with industrial application potential for lowering aflatoxin content in peanuts and peanut products. Food Control.

[19] Shi H., Stroshine R.L., Ileleji K. (2017). Determination of the Relative Effectiveness of Four Food Additives in Degrading Aflatoxin in Distillers Wet Grains and Condensed Distillers Solubles. J. Food Prot..

[20] Guan Y., Chen J., Nepovimova E., Long M., Wu W., Kuca K. (2021). Aflatoxin Detoxification Using Microorganisms and Enzymes. Toxins.

[21] Haskard C.A., El-Nezami H.S., Kankaanpää P.E., Salminen S., Ahokas J.T. (2001). Surface Binding of Aflatoxin B_1_ by Lactic Acid Bacteria. Appl. Environ. Microbiol..

[22] Kolosova A., Stroka J. (2011). Substances for reduction of the contamination of feed by mycotoxins: A review. World Mycotoxin J..

[23] Samuel S.M., Aiko V., Panda P., Mehta A. (2013). Aflatoxin B1 Occurrence, Biosynthesis and its Degradation. J. Pure Appl. Microbiol..

[24] Raksha Rao K., Vipin A.V., Hariprasad P., Anu Appaiah K.A., Venkateswaran G. (2017). Biological detoxification of Aflatoxin B1 by *Bacillus licheniformis* CFR1. Food Control.

[25] Watanakij N., Visessanguan W., Petchkongkaew A. (2020). Aflatoxin B1-degrading activity from *Bacillus subtilis* BCC 42005 isolated from fermented cereal products. Food Addit. Contam. Part A.

[26] Huang L., Duan C., Zhao Y., Gao L., Niu C., Xu J., Li S. (2017). Reduction of Aflatoxin B1 Toxicity by *Lactobacillus plantarum* C88: A Potential Probiotic Strain Isolated from Chinese Traditional Fermented Food “Tofu”. PLoS ONE.

[27] Cai M., Qian Y., Chen N., Ling T., Wang J., Jiang H., Wang X., Qi K., Zhou Y. (2020). Detoxification of aflatoxin B1 by *Stenotrophomonas* sp. CW117 and characterization the thermophilic degradation process. Environ. Pollut..

[28] Fang Q., Du M., Chen J., Liu T., Zheng Y., Liao Z., Zhong Q., Wang L., Fang X., Wang J. (2020). Degradation and Detoxification of Aflatoxin B1 by Tea-Derived *Aspergillus niger* RAF106. Toxins.

[29] Kabak B., Dobson A.D.W., Var I. (2006). Strategies to prevent mycotoxin contamination of food and animal feed: A review. Crit. Rev. Food Sci. Nutr..

[30] Oberoi K., Tolun A., Altintas Z., Sharma S. (2021). Effect of Alginate-Microencapsulated Hydrogels on the Survival of *Lactobacillus rhamnosus* under Simulated Gastrointestinal Conditions. Foods.

[31] Średnicka P., Juszczuk-Kubiak E., Wójcicki M., Akimowicz M., Roszko M.Ł. (2021). Probiotics as a biological detoxification tool of food chemical contamination: A review. Food Chem. Toxicol..

[32] Hu Y., Zhang L., Wen R., Chen Q., Kong B. (2020). Role of lactic acid bacteria in flavor development in traditional Chinese fermented foods: A review. Crit. Rev. Food Sci. Nutr..

[33] Milanda T., Soemarie Y., Barliana M. (2021). Fermented foods as probiotics: A review. J. Adv. Pharm. Technol. Res..

[34] Hernandez-Mendoza A., Garcia H.S., Steele J.L. (2009). Screening of *Lactobacillus casei* strains for their ability to bind aflatoxin B1. Food Chem. Toxicol..

[35] EL-Nezami H., Kankaanpaa P., Salminen S., Ahokas J. (1998). Ability of Dairy Strains of Lactic Acid Bacteria to Bind a Common Food Carcinogen, Aflatoxin B1. Food Chem. Toxicol..

[36] Taheur F.B., Fedhila K., Chaieb K., Kouidhi B., Bakhrouf A., Abrunhosa L. (2017). Adsorption of aflatoxin B1, zearalenone and ochratoxin A by microorganisms isolated from Kefir grains. Int. J. Food Microbiol..

[37] Gratz S., Täubel M., Juvonen R.O., Viluksela M., Turner P.C., Mykkänen H., El-Nezami H. (2006). *Lactobacillus rhamnosus* Strain GG Modulates Intestinal Absorption, Fecal Excretion, and Toxicity of Aflatoxin B1 in Rats. Appl. Environ. Microbiol..

[38] Zhu Y., Xu Y., Yang Q. (2021). Antifungal properties and AFB_1_ detoxification activity of a new strain of *Lactobacillus plantarum*. J. Hazard. Mater..

[39] Mosallaie F., Jooyandeh H., Hojjati M., Fazlara A. (2019). Biological reduction of aflatoxin B1 in yogurt by probiotic strains of *Lactobacillus acidophilus* and *Lactobacillus rhamnosus*. Food Sci. Biotechnol..

[40] Markov K., Frece J., Pleadin J., Bevardi M., Barisic L., Kljusuric J.G., Vulic A., Jakopovic Z., Mrvcic J. (2019). *Gluconobacter oxydans*—Potential biological agent for binding or biotransformation of mycotoxins. World Mycotoxin J..

[41] Pourmohammadi K., Sayadi M., Abedi E., Mousavifard M. (2022). Determining the adsorption capacity and stability of Aflatoxin B1, Ochratoxin A, and Zearalenon on single and co-culture *L. acidophilus* and *L. rhamnosus* surfaces. J. Food Compos. Anal..

[42] Abrunhosa L., Paterson R., Venâncio A. (2010). Biodegradation of Ochratoxin A for Food and Feed Decontamination. Toxins.

[43] Sangare L., Zhao Y., Folly Y.M.E., Chang J., Li J., Selvaraj J.N., Xing F., Zhou L., Wang Y., Liu Y. (2015). Aflatoxin B_1_ Degradation by a *Pseudomonas* Strain. Toxins.

[44] Shu X., Wang Y., Zhou Q., Li M., Hu H., Ma Y., Chen X., Ni J., Zhao W., Huang S. (2018). Biological Degradation of Aflatoxin B_1_ by Cell-Free Extracts of *Bacillus velezensis* DY3108 with Broad PH Stability and Excellent Thermostability. Toxins.

[45] Bueno D.J., Casale C.H., Pizzolitto R.P., Salvano M.A., Oliver G. (2007). Physical adsorption of aflatoxin B_1_ by lactic acid bacteria and *Saccharomyces cerevisiae*: A theoretical model. J. Food Prot..

[46] Yang X., Teng D., Wang X., Guan Q., Mao R., Hao Y., Wang J. (2016). Enhancement of Nutritional and Antioxidant Properties of Peanut Meal by Bio-modification with *Bacillus licheniformis*. Appl. Biochem. Biotechnol..

[47] Li S., Li C., Chen S., Wang X., Liu J., Deng X., Cai H., Liu G. (2023). Effects of Solid-State Fermentation on the Standardized Ileal Digestibility of Amino Acids and Apparent Metabolizable Energy in Peanut Meal Fed to Broiler Chickens. Fermentation.

[48] Shao S., Cai J., Du X., Wang C.G., Lin J.G., Dai J. (2016). Biotransformation and detoxification of aflatoxin B_1_ by extracellular extract of *Cladosporium uredinicola*. Food Sci. Biotechnol..

[49] Wang Y., Zhao C.X., Zhang D.D., Zhao M.M., Zheng D., Lyu Y.C., Cheng W., Guo P., Cui Z.J. (2017). Effective degradation of aflatoxin B_1_ using a novel thermophilic microbial consortium TADC7. Bioresour. Technol..

[50] Xie Y.L., Wang W., Zhang S.J. (2019). Purification and identification of an aflatoxin B_1_ degradation enzyme from *Pantoea* sp. T6. Toxicon.

[51] Xu Y., Zhao R., Liu C. (2023). Degradation of Aflatoxin B_1_ in Moldy Maize by *Pseudomonas aeruginosa* and Safety Evaluation of the Degradation Products. Foods.

[52] NPC (National Pharmacopoeia Commission) (2020). Chinese Pharmacopoeia.

[53] (2016). National Standard for Food Safety: Determination of Protein Content in Food.

[54] (2016). National Standard for Food Safety: Determination of Amino Acid Content in Food.

[55] Liu M., Wang X., Zhang Y., Xu L., Liu Y., Yu L., Ma F., Wang X., Gong Z., Zhang L. (2023). Chemical composition of walnuts from three regions in China. Oil Crop Sci..

[56] Qiu T., Wang H., Zhu Y., Yang Y., Ji J., Sun X. (2020). Optimization of Microbial Fermentation Removal of Aflatoxins in Peanut Meal by *Aspergillus niger* Using Response Surface Methodology. Farm Prod. Process..

