# Supplementary material for: Lactiplantibacillus plantarum YS-718 Probiotics Screened from Traditional Chinese Fermented Vegetables for Aflatoxin B1 Removal
Source: Toxins (Basel). 2026 Jun 23;18(7):275. doi: 10.3390/toxins18070275 (PMC13417330; doi:10.3390/toxins18070275)
Supplement: Supplementary file 1 [file toxins-18-00275-s001.zip › toxins-4337821-supplementary.pdf]

Supplementary Materials: *Lactiplantibacillus plantarum* YS-718 probiotics screened from traditional Chinese fermented vegetables for aflatoxin B_1 removal

Fang Yuan, Guofeng Chen, Xianglong Yang, Ling Cheng, Qi Zhang, Peiwu Li, Baohai Liu and Jin Mao

This supplementary material contains 2 figures and 1 table.

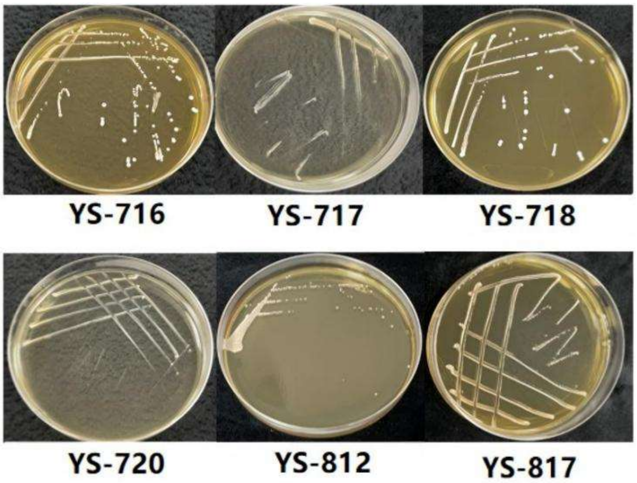

Figure S1. The photographs of the six screened strains using coumarin as the sole carbon source.

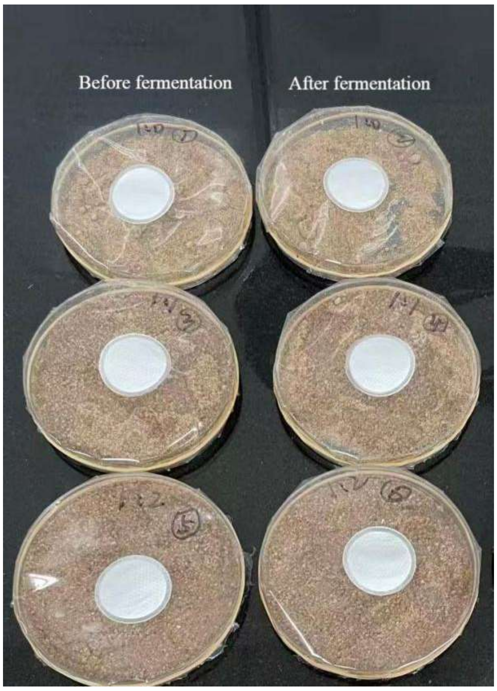

Figure S2. The photographs of peanut meal samples before and after treatment/fermentation.

Table S1. The identification information of *Lactiplantibacillus plantarum* YS-718.

Gene sequence	CCTGTACGACTTCACCCTAATCATCTGTCCCAC-<br>CTTAGGCGGCTGGTTCCTAAAAGGTTACCCCACCGACTTTGGGTGTTACAAA<br>CTCTCATGGTGTGACGGGCGGTGTGTACAAGGCCCGGGAACGTATTCAC-<br>CGCGG-<br>CATGCTGATCCGCGATTACTAGCGATTCCGACTTCATGTAGGCGAGTTGCAG<br>CCTACAATCCGAACTGAGAATGGCTTTAAGAGATTAGCTTACTCTCGCGAG-<br>TTCG-<br>CAACTCGTTGTACCATCCATTGTAGCACGTGTGTAGCCCAGGTCATAAGGGG<br>CATGATGATTTGACGTCATCCCCACCTTCCTCCGGTTTGTCACCGGCAG-<br>TCTCACCAGAG-<br>TGCCCAACTTAATGCTGGCAACTGATAATAAGGGTTGCGCTCGTTGCGGGA<br>CTTAACCCAACATCTCACGACACGAGCTGACGACAACCATGCACCACCTG-<br>TATCCATGTCCCCGAAGGGAACGTCTAATCTCTTAGATTTGCATAGTATGTC<br>AAGACCTGGTAAGGTTCTTCGCGTAGCTTCGAATTAAACCACATGCTCCAC-<br>CGCTT-<br>GTGCGGGCCCCCGTCAATTCCTTTGAGTTTCAGCCTTGCGGCCGTACTCCCC<br>AGGCGGAATGCTTAATGCGTTAGCTGCAGCACTGAAGGGCG-<br>GAAACCCTCCAACACTTAG-<br>CATTCATCGTTTACGGTATGGACTACCAGGGTATCTAATCCTGTTTGCTACC<br>CATACTTTCGAGCCTCAGCGTCAGTTACAGACCAGA-<br>CAGCCGCCTTCGCCAC-<br>TGGTGTTCTTCCATATATCTACGCATTTCACCGCTACACATGGAGTTCCACT<br>GTCCTCTTCTGCACTCAAGTTTCCCAGTTTCCGATGCACTTCTTCGGTT-<br>GAGCCGAAGGCTTTCACATCAGACTTAAAAAACCGCCTGCGCTCGCTTTAC<br>GCCCAATAAATCCGGACAACGCTTGCCACCTACGTATTAC-<br>CGCGGCTGCTGGCACGTAG-<br>TTAGCCGTGGCTTTCTGGTTAAATACCGTCAATACCTGAACAGTTACTCTCA<br>GATATGTTCTTCTTTAACAACAGAGTTTTACGAGCCGAAACCCTTCTTCAC-<br>TCAC-<br>GCGGCGTTGCTCCATCAGACTTTCGTCCATTGTGGAAGATTCCCTACTGCTG<br>CCTCCCGTAGGAGTTTGGGCCGTGTCTCAGTCCCAATGTGGCCGAT-<br>TACCCTCTCAGGTCGGCTACGTATCATTGCCATGGTGAGCCGTTACCCCACC<br>ATCTAGCTAATACGCCGCGGGACCATCCAAAAGTGATAGCCGAA-<br>GCCATCTTTCAAACTCG-<br>GACCATGCGGTCCAAGTTGTTATGCGGTATTAGCATCTGTTTCCAGGTGTTA<br>TCCCCCGCTTCTGGGCAGGTTTCCCACGTGTTACTCACCAGTTCGCCAC-<br>TCAC-<br>TCAAATGTAAATCATGATGCAAGCACCAATCAATACCAGAGTTCGTTCGAC<br>TTGCATGTATTAGGCACGCCGCA
Strain type	*Lactiplantibacillus plantarum*
Homology values	100% with *Lactiplantibacillus plantarum* (KR153313.1)
Accession number in Chinese Typical Cultures Depository Center (CCTCC)	CCTCC M 20232226
